# Illusory early-warning signals of Greenland Ice Sheet destabilization

**DOI:** 10.1073/pnas.2623190123

**Published:** 2026-09-08

**Authors:** Alexander A. Robel, Grant Holly, Aminat A. Ambelorun

**Affiliations:** ^a^ School of Earth and Atmospheric Sciences, Georgia Institute of Technology, Atlanta, GA 30318

**Keywords:** ice sheets, destabilization, sea Level, early-warning signals

## Abstract

Interannual variability of surface mass balance over the Greenland Ice Sheet has been increasing, which has been cited as an early-warning signal of ice sheet destabilization under current climatic conditions. A destabilized ice sheet would continue to lose mass and contribute to global sea level rise, even if climate stopped changing in the future. Here, we investigate the mechanisms driving nonstationarity of surface mass balance variability in a high-fidelity surface mass balance reconstruction, and a calibrated simple model. We show that, regardless of the proximity to destabilization, increasing variability in surface mass balance is an expected consequence of atmospheric warming on low-accumulation parts of the ice sheet. Autocorrelation of surface mass balance exhibits spurious multidecadal trends under a wide range of climatic conditions due to the slow response of ice sheets to climatic fluctuations, making this a poor early-warning signal of destabilization. Though these findings do not rule out the potential for destabilization under current or future climate, they provide a conceptual framework for understanding the drivers of surface melt variability.

A thinning ice sheet can enter into a runaway acceleration of surface melting due to the atmosphere being warmer at lower altitudes (1). The climate conditions necessary for such ice sheet destabilization are highly uncertain, with some models indicating destabilization has already occurred and others projecting many centuries of continued stability under future warming (2, 3). Recent work (4) hypothesized that increasing interannual variance and lag-1 autocorrelation of Greenland SMB as recorded in shallow ice cores (5) over the industrial era are strong “early-warning signals” (EWS) for the destabilization of the western part of the Greenland Ice Sheet at temperatures already observed in this region. Satellite observations (6) and observationally constrained surface mass balance (SMB) models (7, 8) also exhibit increasing interannual variability in Greenland SMB and runoff since at least 2000. Projections indicate SMB variability will continue to increase into the future with atmospheric warming. Here, using a high-resolution historical SMB reconstruction with ice sheet geometry that is fixed in time and a simple ice sheet mass balance model, we show that recent trends in the statistics of SMB variability are not reliable EWS of ice sheet destabilization.

## Results and Discussion

We examine trends in both the variance and lag-1 autocorrelation coefficient of interannual SMB over the Greenland Ice Sheet in the Modèle Atmosphérique Régional (MAR) v3.14 forced by the ERA5 climate reanalysis product. MAR is a high-resolution (5 to 10 km) SMB model that has been extensively validated against observations and other high-fidelity SMB models (9). Importantly, the ice sheet surface elevation in MAR is static in time, so changes in SMB can only be the result of large-scale climatic changes in the ERA5 reanalysis product and snow, firn and melt processes, rather than the SMB-elevation feedback.

**Fig. 1.**
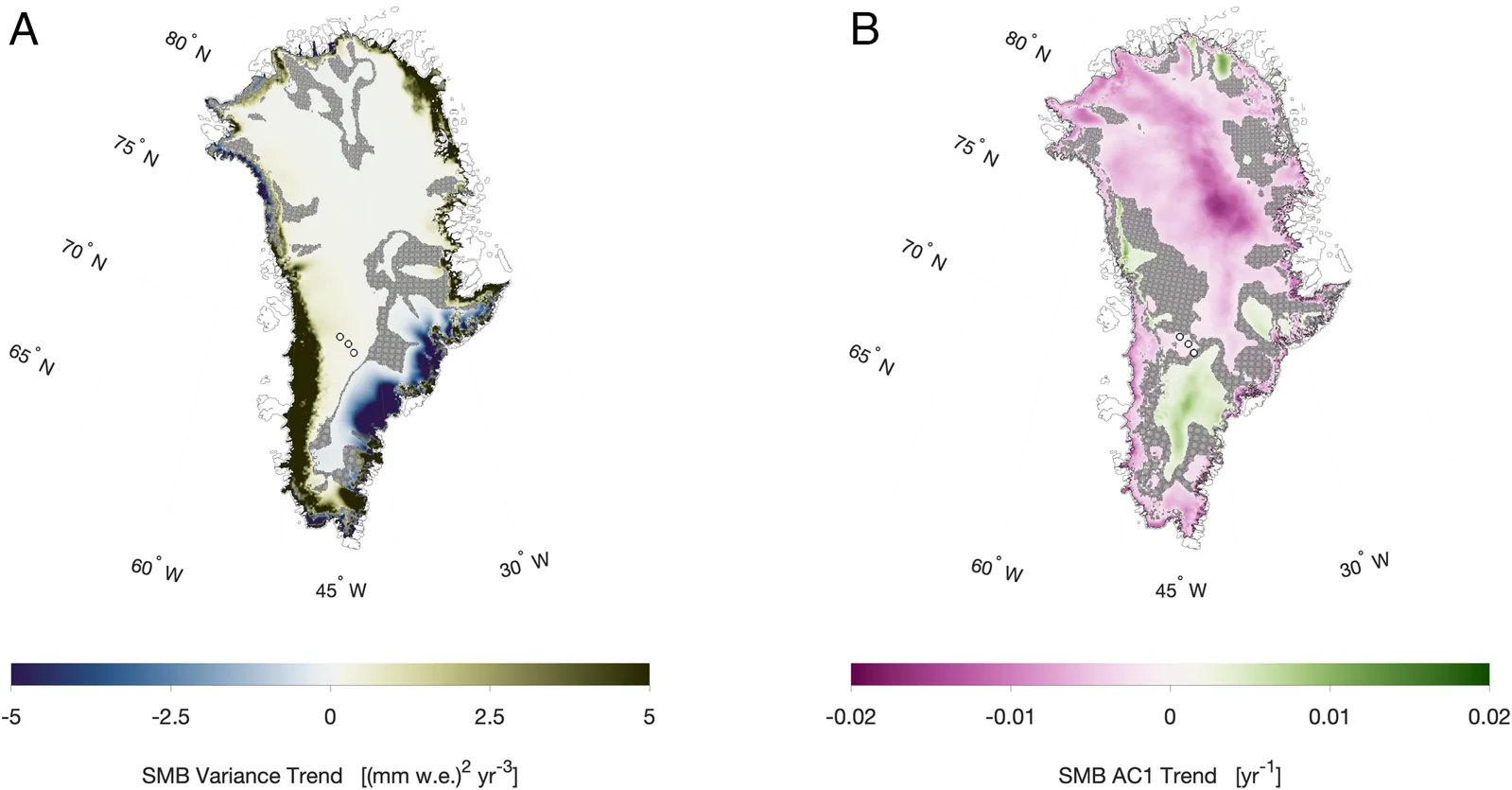
Maps of trends in Greenland SMB interannual variability from MAR v3.14 over 1940–2025 (20-y trailing window). (*A*) Variance trend. (*B*) Lag-1 autocorrelation (AC1) trend. Hatching indicates where zero trend cannot be ruled out at 95% CI. Three circles are locations of ice cores in Trusel et al. (5).

Trends in interannual SMB variance and lag-1 autocorrelation (AC1) derived from the MAR historical reconstruction over 1940–2015 are plotted in Fig. 1. Variance is increasing over most (63%) of the ice sheet area, with small areas (16%) in the Southeast and Northwest exhibiting decreasing variance and some parts (21%) of the ice sheet interior experiencing no change. AC1 shows decidedly different trends, with decreasing AC1 over most (57%) of the ice sheet area, increasing AC1 over a small area (12%) primarily in the Southeast, and no statistically distinguishable trend over a substantial (31%) area.

The finding of increasing SMB variance over most of the ice sheet is consistent with prior analyses from both observations and other SMB models (4, 6–8, 10), even without the destabilizing SMB-elevation feedback. Increasing SMB variability has been shown to be caused by lengthening of the bare-ice season, where lowered albedo leads to increased sensitivity to variability in incoming shortwave radiation (7, 10). In contrast, Southeast Greenland is a region where accumulation and melt are both high and increasing over the historical period (9), so the bare-ice season does not lengthen, leading to decreasing or nonsignificant variance trends.

Decreasing or unchanged AC1 over most of the ice sheet has no established explanation in prior analyses. Notably, we find evidence for decreasing AC1 in West Greenland including the locations of ice cores where previous analysis (4) found increasing AC1 for a normalized and log-transformed metric of melt. AC1 trends lack the same obvious spatial correlation to SMB as variance trends. Thus, we seek an approach that will explain these trends.

We consider a simple and physically motivated model of ice sheet mass balance:

[1] $$\begin{array}{c} \frac{\partial \Delta h}{\partial t}=B\left(T_{s}\right)+f\left(\Delta h\right). \end{array}$$

$B(T_{s})$ is the SMB as a function of ice sheet surface temperature $T_{s}=T_{\mathrm{ff}}-\Gamma \Delta h$, where $T_{\mathrm{ff}}$ is far-field atmospheric temperature, Δh is ice sheet thickness change, and Γ is atmospheric lapse rate. The lapse rate effect captured in −ΓΔh causes ice sheet destabilization through the SMB-elevation feedback. Far-field temperature is constrained using station data from Central West Greenland over summer months (available from ref. 5) with the function $T_{\mathrm{ff}}=T_{0}+at+T^{\prime }\left(t\right)$, where $T_{0}$ is the initial mean temperature, a is the long-term rate of temperature increase, and $T^{\prime }\left(t\right)$ are zero-mean random interannual fluctuations in temperature.

Recent observational studies (5, 10) provide strong evidence for a purely exponential relationship between surface melt rates and temperature, $B\left(T_{s}\right)=-\beta \exp\left(\lambda T_{s}\right)$, where β and λ have been empirically constrained from observations and models (5). One might consider adding a constant to $B(T_{s})$ to represent snowfall accumulation at low surface temperatures, however at the range of temperatures considered in this study (well above 0 ^°^C) such a term has a negligible effect on total SMB.

f(Δh) represents “dynamic thinning,” the contribution of ice flow to local mass balance. Following established theories of glacier mass balance (11), we represent dynamic thinning as a linear restoring term, f(Δh)=αΔh, with negative α. At warm surface temperatures, the SMB term $B(T_{s})$ causes rapid thinning and can lead to ice sheet destabilization through the SMB-elevation feedback. However, if f(Δh) is sufficiently large, it balances $B(T_{s})$ and maintains a stable ice sheet steady state. Altimetry observations and SMB reconstructions constrain the magnitude of α to be $O\left(10^{-1}\right)\;y^{-1}$ (*SI Appendix*). With these constraints on the magnitude, we consider a range of α values, while also tuning it in individual simulations to match observed rates of Greenland Ice Sheet thinning.

**Fig. 2.**
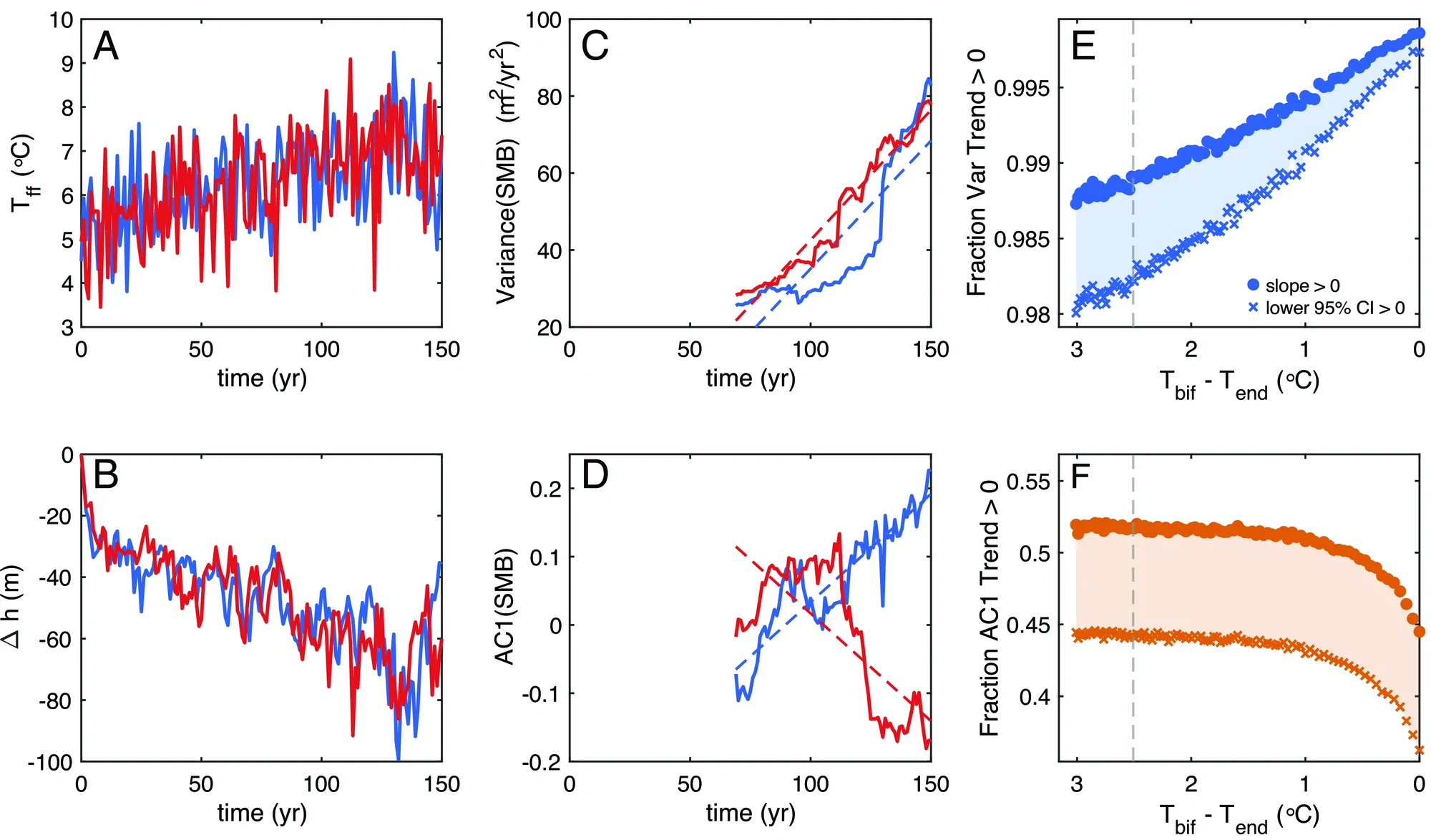
SMB variability in a simple model under atmospheric warming. (*A*) Two random 150-y realizations of forcing $T_{\mathrm{ff}}$. (*B*) Modeled Δh, ice thickness change, under $T_{\mathrm{ff}}$ forcing, $\alpha =0.4\;y^{-1}$. (*C* and *D*) Rolling variance and AC1 of detrended SMB in a 70-y trailing window. Dashed lines are ordinary least squares fits. (*E* and *F*) Fraction of simulations with positive variance and AC1 trends versus the distance to the destabilization temperature. Filled circles and crosses are positive trend fraction using central estimate and lower 95% CI bound on slope. The dashed vertical line marks α used in (*A*–*D*).

Two simulations with this simple model differing only in the realization of random temperature forcing (Fig. 2*A*) both show rapid thinning (Fig. 2*B*) and ∼150% increases in SMB variance (Fig. 2*C*), consistent with observations (6, 12). However, these two simulations have significant trends in lag-1 autocorrelation (AC1) of opposite sign (Fig. 2*D*).

We characterize model behavior over a wide range of α values corresponding to stable states up to the ice sheet destabilization point. For each value of α, we run $10^{5}$ simulations with different random realizations of $T^{\prime }\left(t\right)$. Trends in SMB variance are significantly positive in more than 98% of simulations (Fig. 2*E*), even when the atmospheric temperature is far from the point of destabilizing the ice sheet. This is an unavoidable consequence of $\partial^{2}B/\partial T_{s}^{2}>0$, which requires that increasing mean $T_{s}$ produces increasing SMB variance with the same $T_{s}$ variability.

Conversely, ∼50% of simulations have a positive trend in lag-1 autocorrelation (45% if restricted to significant trends) across all α values considered (Fig. 2*F*). The slow (∼decades) response of ice sheets to random forcing produces multidecadal fluctuations in AC1 that can be spuriously identified as trends over subcentennial time scales (13). Approaching ice sheet destabilization decreases the fraction of simulations with positive AC1 trends, the opposite of EWS theory. This counterintuitive result can be understood by restating the lag-1 autocorrelation as

[2] $$\begin{array}{c} \rho_{1}=\frac{\text{Cov}(\Delta h_{t},\Delta h_{t-1})}{\sqrt{\text{Var}\left(\Delta h_{t}\right)\text{Var}\left(\Delta h_{t-1}\right)}}=\mu \sqrt{\frac{\text{Var}(\Delta h_{t-1})}{\text{Var}(\Delta h_{t})}}, \end{array}$$

where μ is the restoring tendency of the system. As the system approaches destabilization, μ→1, which increases $\rho_{1}$. However, if variance increases at an accelerating rate, $\sqrt{\frac{\text{Var}(\Delta h_{t-1})}{\text{Var}(\Delta h_{t})}}$ decreases, which decreases $\rho_{1}$, as seen in our simulations and MAR (Fig. 1*B*).

We conclude that trends in variance and lag-1 autocorrelation of SMB are not reliable EWS of ice sheet destabilization. While these results do not rule out the potential for future destabilization, they do indicate that other approaches are necessary to determine when destabilization may occur. Ice sheet models calibrated to observations can make predictions about the stability of the Greenland Ice Sheet under current and future climate (2, 3). However, such approaches currently produce substantial disagreement on when destabilization may occur, and thus considerable further effort will be needed to reduce model-based projection uncertainty.

Increasing variability of SMB at a range of time scales may have other consequences for the dynamic ice sheet system, including melt refreezing within firn, increased water pressure in the subglacial drainage system, and frontal ablation through meltwater discharge. Representing nonstationarity in SMB and surface runoff in models is thus imperative to accurately project future ice sheet change.

## Materials and Methods

To analyze trends in MAR SMB interannual variability, we compute 20-y trailing variance and lag-1 autocorrelation, and fit OLS linear trends. We esti mate α from Eq. **1** using ICESat/ICESat-2 data and MARv3.14 SMB data. The simple-model is integrated with the forward Euler method with one-year time steps and draws from a Gaussian distribution for temperature fluctuations. Full details are in *SI Appendix*.

## Supplementary Material

Appendix 01 (PDF)

## Data Availability

All code used in this study are archived in a GitHub repository (14). MAR v3.14 runs are available from (15).
