# Supplementary material for: Illusory early-warning signals of Greenland Ice Sheet destabilization
Source: Proc Natl Acad Sci U S A. 2026 Sep 8;123(37):e2623190123. doi: 10.1073/pnas.2623190123 (PMC13578335; doi:10.1073/pnas.2623190123)
Supplement: Supplementary file 1 [file pnas.2623190123.sapp.pdf]

Supporting Information for

Illusory Early-Warning Signals of Greenland Ice Sheet Destabilization

Alexander A. Robel, Grant Holly, Aminat A. Ambelorun

[1]**To whom correspondence should be addressed. E-mail: robeleas.gatech.edu**

This PDF file includes:

Supporting text
SI References

Supporting Information Text

Processing and calculating trends for MAR SMB statistics

We analyze annual MAR v3.14 SMB from 1940-2025 on the native $\sim$1 km polar-stereographic grid. We first calculate annual SMB averages from the monthly data to focus on interannual variability. For each ice-covered cell (MAR mask category ≥ 4) we compute a 20-yr trailing rolling variance and a 20-yr trailing rolling lag-1 autocorrelation (best-linear-unbiased estimator) of annual SMB. Linear trends in each rolling series are fit by ordinary least squares (OLS); two-sided 95% confidence intervals on the slope are computed from the residual standard error using Student's t distribution with $n-2$ degrees of freedom. Cells whose slope CI overlaps zero are stippled in Fig. 1. Reported area fractions are ice-cell counts on the MAR grid.

Estimating α from observations

We estimate α from ICESat-1/2 satellite altimetry and MAR v3.14 SMB reconstruction. Integrating Eq. 1 over the period $T = 17\,\mathrm{yr}$ (2003–2019) gives $\Delta h = \int_0^T B\,dt + \alpha \int_0^T h\,dt$. Assuming the elevation anomaly grew approximately linearly from zero to Δh over the window gives $\int_0^T h\,dt \approx \Delta h\,T/2$ and the estimate

$$\alpha \;\approx\; \frac{\Delta h \;-\; \int_0^T B\,dt}{\Delta h \cdot T/2}. \qquad [1]$$

The scale of values found through this approach sets the α sweep range used in Fig. 2e,f. Other destabilizing feedbacks related to ice sheet discharge may cause α to be positive, though such feedbacks are not the focus of this study and so we do not consider such cases.

Greenland ice thinning rates are available from: https://digital.lib.washington.edu/researchworks/handle/1773/45388, the repository associated with (1).

Analytical linear stability analysis of simple model

Consider the deterministic ($T' \equiv 0$) simple model: $\dot{\Delta h} = f(\Delta h, T_{ff})$ with $f \equiv b_0 - \beta\, e^{\lambda(T_{ff} - \Gamma\Delta h)} + \alpha\Delta h$. Fixed points $\Delta h_*(T_{ff})$ satisfy $f = 0$, which has a closed-form solution in terms of the Lambert W function,

$$\Delta h_*(T_{ff}) \;=\; \frac{1}{\lambda\Gamma}\, W\!\left(\frac{\beta\lambda\Gamma}{\alpha}\, e^{\lambda T_{ff}}\right). \qquad [2]$$

The principal branch W_0 gives the stable upper fixed point that we are interested in, which disappears at the bifurcation point.

The linear stability (eigenvalue) of the fixed point Δh_* is

$$\omega(\Delta h_*, T_{ff}) \;=\; \left.\frac{\partial f}{\partial \Delta h}\right|_{\Delta h_*} \;=\; \beta\lambda\Gamma\, e^{\lambda T_s} + \alpha. \qquad [3]$$

A bifurcation occurs where two co-existing fixed points coalesce and linear stability is lost; it is given by $f = 0$ and $\omega = 0$ simultaneously. The latter yields $e^{\lambda T_s} = -\alpha/(\beta\lambda\Gamma)$, and substituting into $f = 0$ gives the bifurcation point,

$$\Delta h_*(\alpha) = -\frac{1}{\lambda\Gamma}, \qquad T_{\mathrm{bif}}(\alpha) = \frac{1}{\lambda}\left[\ln\frac{-\alpha}{\beta\lambda\Gamma} - 1\right]. \qquad [4]$$

The horizontal axis of Fig. 2e,f is $T_{\mathrm{diff}} = T_{\mathrm{bif}}(\alpha) - T_{\mathrm{end}}$ with $T_{\mathrm{end}} = T_0 + a\,t_f$.

Simple model parameters and integration

The simple model equation is integrated using the forward Euler method on a $\Delta t = 1\,\mathrm{yr}$ time step over 150 yr from $\Delta h(t=0) = 0$. The SMB parameters $\beta = 1.307\,\mathrm{m\,yr^{-1}}$ and $\lambda = 0.3955\,(^\circ\mathrm{C})^{-1}$, are taken from (2), the initial mean far-field temperature is $T_0 = 5.3\,^\circ\mathrm{C}$, taken from (3). In polar regions, the atmospheric lapse rate is observed to be approximately 6 °C/km (4). In Greenland a representative rate of snowfall (b_0) achieved at low atmospheric temperatures in the ice sheet interior is 0.3 m/yr. Temperature station data (2) from just off the Greenland ice sheet is used to set the far-field temperature trends and variability: $a = 1.3$ °C/century and the standard deviation of T' at 0.9 °C.

The interannual noise $T'(t)$ is sampled independently at each time step with $\sigma_{T'}\sqrt{\Delta t}$ scaling, so that its per-year variance is independent of Δt. Before computing the rolling statistics in Fig. 2c,d, SMB is detrended over each 70-yr window to remove the imposed warming trend. Dashed lines in Fig. 2c,d are OLS linear fits to the portion of the windowed record following the initial 70 years of the record required for the trailing window.

For Fig. 2e,f we sweep 100 values of α from a comfortably stable end ($T_{\mathrm{diff}} = T_{\mathrm{bif}}(\alpha) - T_{\mathrm{end}} \approx 3\,^\circ\mathrm{C}$) down to the saddle-node bifurcation ($T_{\mathrm{diff}} = 0$), with 10^5 independent realizations at each α. $T_{\mathrm{bif}}(\alpha)$ is the analytical saddle-node fold of the deterministic system ($T' \equiv 0$), obtained by solving $f(h, T_{ff}) = 0$ and $\partial f/\partial h = 0$ simultaneously on the warm-melt branch, where $f(h, T_{ff}) \equiv B(T_s) + \alpha h$. Detection rates are computed from per-realization OLS on the rolling EWS: filled circles are the fraction of realizations with positive central slope; crosses are the fraction whose lower 95% CI bound on the slope exceeds zero.

References

1. B Smith, et al., Pervasive ice sheet mass loss reflects competing ocean and atmosphere processes. *Science* **368**, 1239–1242 (2020).
2. LD Trusel, et al., Nonlinear rise in Greenland runoff in response to post-industrial Arctic warming. *Nature* **564**, 104–108 (2018).
3. N Boers, M Rypdal, Critical slowing down suggests that the western Greenland Ice Sheet is close to a tipping point. *Proc. Natl. Acad. Sci.* **118**, e2024192118 (2021).
4. RS Fausto, AP Ahlstrøm, D Van As, CE Bøggild, SJ Johnsen, A new present-day temperature parameterization for Greenland. *J. Glaciol.* **55**, 95–105 (2009).
